# Ultrasound-Assisted Maillard Conjugation of Yeast Protein Hydrolysate with Polysaccharides for Encapsulating the Anthocyanins from Aronia

**DOI:** 10.3390/antiox13050570

**Published:** 2024-05-05

**Authors:** Loredana Dumitrașcu, Mihaela Brumă (Călin), Mihaela Turturică, Elena Enachi, Alina Mihaela Cantaragiu Ceoromila, Iuliana Aprodu

**Affiliations:** 1 Faculty of Food Science and Engineering, Dunarea de Jos University of Galati, Domnească Street 111, 800201 Galați, Romania; loredana.dumitrascu@ugal.ro (L.D.); mihaela.calin@ugal.ro (M.B.); mihaela.turturica@ugal.ro (M.T.); elena.enachi@ugal.ro (E.E.); 2 Faculty of Medicine and Pharmacy, Dunarea de Jos University of Galati, 35 A.I. Cuza Str., 800010 Galaţi, Romania; 3 Cross-Border Faculty, Dunarea de Jos University of Galati, Domnească Street 111, 800201 Galaţi, Romania; alina.cantaragiu@ugal.ro

**Keywords:** ultrasound, Maillard, yeast protein hydrolysate, encapsulation, anthocyanins

## Abstract

Valorisation of food by-products, like spent brewer’s yeast and fruit pomaces, represents an important strategy for contributing to sustainable food production. The aims of this study were to obtain Maillard conjugates based on spent yeast protein hydrolysate (SYH) with dextran (D) or maltodextrin (MD) by means of ultrasound treatment and to use them for developing encapsulation systems for the anthocyanins from aronia pomace. The ultrasound-assisted Maillard conjugation promoted the increase of antioxidant activity by about 50% compared to conventional heating and SYH, and was not dependent on the polysaccharide type. The ability of the conjugates to act as wall material for encapsulating various biologically active compounds was tested via a freeze-drying method. The retention efficiency ranged between 58.25 ± 0.38%–65.25 ± 2.21%, while encapsulation efficiency varied from 67.09 ± 2.26% to 88.72 ± 0.33%, indicating the strong effect of the carrier material used for encapsulation. The addition of the hydrolysed yeast cell wall played a positive effect on the encapsulation efficiency of anthocyanins when used in combination with the SYH:MD conjugates. On the other hand, the stability of anthocyanins during storage, as well as their bioavailability during gastrointestinal digestion, were higher when using the SYH:D conjugate. The study showed that hydrolysis combined with the ultrasound-assisted Maillard reaction has a great potential for the valorisation of spent brewer’s yeast as delivery material for the encapsulation of bioactive compounds.

## 1. Introduction

Spent brewer’s yeast (SBY) is a by-product resulting from beer production, accounting for about 15% of the total by-products generated by this industry [1]. SBY has an attractive nutritional composition because of the presence of high amounts of high-quality proteins (up to 60%), carbohydrates (up to 35%), lipids, vitamins, fibres, and minerals [2]. Unlike other by-product sources, SBY is available throughout the year, and the valorisation by developing innovative value-added products can contribute to the transition from the linear to circular bioeconomy. Although yeast material was used successfully for encapsulation of both hydrophilic and hydrophobic food compounds, the potential of SBY was not fully explored [3,4,5]. Many previous studies used pure baker’s yeasts as wall material; however, the composition of spent yeasts derived from the brewing and wine industry is different, influencing the material characteristics [3]. In addition, preliminary yeast treatments, such as plasmolysis or enzymatic hydrolysis, applied before encapsulation, have a significant impact on the final characteristics of the delivery material. 

The Maillard reaction is a covalent glycation strategy for producing protein–polysaccharide conjugates, and one of the most efficient methods for improving proteins functionality in terms of emulsifying, foaming, solubility, and antioxidant properties [6,7]. These properties are promoted when the Maillard reaction is performed under controlled conditions, regarding reactants characteristics, ratio between reactants, reaction temperature, and pH. The use of polysaccharides in the Maillard reaction has the advantage of providing large steric hindrance, an important feature of proteins with high functionality [7]. Moreover, the abovementioned properties make the Maillard conjugates promising delivery systems for various sensitive compounds such as volatile oils or bioactive compounds [8]. Zhou et al. [9] and Jang and Koh [10] indicated that protein–polysaccharides conjugates provide superior protection for encapsulated bioactive compounds during processing, storage, and digestion. The effect of the Maillard reaction of hydrolysed SBY cells with maltodextrin (MD) was recently reported for obtaining an encapsulating material for ascorbic acid [3], while Fu et al. [11] produced glycated yeast cell via the Maillard reaction for delivery of curcumin. Although it contains significant amounts of proteins and total sugars, the potential of soluble proteins of spent yeast hydrolysate as a delivery material has not been tested so far. In recent years, alternative treatments have demonstrated the huge potential for replacing or complementing the conventional heating due to their ability of enhancing the functionality of proteins while limiting the formation of toxic compounds. Among them, ultrasound treatment (US) is one of the most cost-effective methods for promoting and accelerating the Maillard conjugation [6]. However, the use of ultrasound and the Maillard reaction for improving the anthocyanins encapsulation potential of milk proteins has been reported recently by [7]. In a previous study performed by our research group, we tested the effect of several enzymes for producing hydrolysates from SBY with increased antioxidant activity [2]. Thus, our interest was to investigate further the potential of peptides with increased antioxidant activity from SBY for producing Maillard conjugates that can be used as carrier materials for anthocyanins from *Aronia melanocarpa* (black chokeberries) pomace. Black chokeberry (BC) pomace contains important amounts of bioactive compounds, such as anthocyanins, with cyanidin-3-*O*-galactoside, cyanidin-3-*O*-glucoside, cyanidin-3-*O*-arabinoside, and cyanidin-3-*O*-xyloside being the most predominant [12]. Although these compounds exhibit many health-promoting properties, they can be easily prone to degradation, thus encapsulation represents a good alternative for providing target protection [10]. Therefore, the objective of this study was to develop an encapsulation system for anthocyanins from aronia pomace based on spent yeast protein hydrolysate (SYH) and dextran or maltodextrin conjugates by using US-assisted Maillard reactions. First, the effect of US time and polysaccharide type on producing Maillard conjugates with superior antioxidant activity was investigated in comparison with conventional heating. Then, conjugates possessing high antioxidant activity were tested as coating material for anthocyanins from Aronia pomace.

## 2. Materials and Methods

### 2.1. Materials

Dried SBY (42% protein) was provided by a beer factory from Ploiesti, Romania, in 2022. Dextran with an average molecular weight of 40 kDa was purchased from Sigma Aldrich Co. (St. Louis, MO, USA), and Maltodextrin 12 was purchased from Thermo Scientific (Shanghai, China). Fresh black chokeberries (BC) were kindly provided by a local producer (Tătaru, Braila, Romania) in August 2022. The fruits were rinsed with water and stored at −20 °C until they were needed for analysis. All other reagents were analytical grade. 

### 2.2. Preparation of Spent Yeast Protein Hydrolysate (SYH)

**Figure 1:**
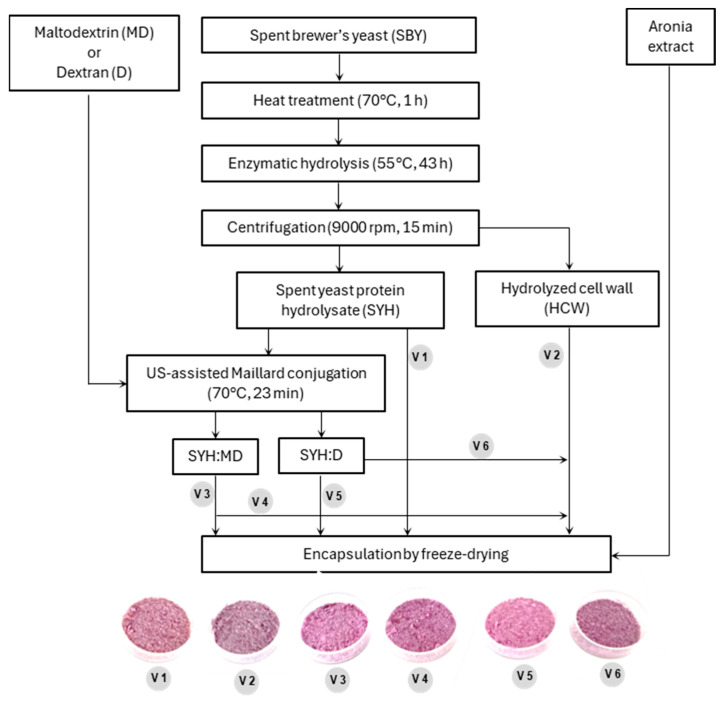
The schematic representation of the experimental plan for the production of microparticles based on spent yeast Maillard conjugates.

SYH was obtained as reported by our research group in a previous study [2]. Briefly, SBY suspension of 12% (*w*/*w*) concentration (pH 7.0) was hydrolysed with Neutrase (1%, based on the solid content) for 43 h at 55 °C. At the end of hydrolysis, the sample had a hydrolysis degree of about 26% and a soluble protein content of about 86 g/100 g dry weight. Then, the sample was centrifuged, and the resulting supernatant (SYH) and hydrolysed cell wall (HCW) were freeze dried separately and stored under refrigeration until use (Figure 1). 

### 2.3. Preparation of Spent Yeast Hydrolysate-Based Conjugates

Lyophilized SYH, dextran (D), and maltodextrin (MD) were rehydrated in phosphate buffer (0.01 mol/L, pH 7.0) and stored at 4 °C overnight to ensure full hydration. Then, SYH (20 mg/mL) was mixed with either D (20 mg/mL) or MD (20 mg/mL), at a ratio of 1:1 (*v*/*v*). The ratio was chosen based on preliminary tests used for identification of the appropriate ratio of reactants for performing the Maillard reaction (results not shown). The resulting slurry (50 mL) was exposed to sonication at 70 °C ± 2 °C at 40% amplitude in pulse mode (5 s on and 5 s off) for 5 to 23 min by using a VCX 500 ultrasound system with total power of 500 W and frequency of 20 kHz (Sonics Vibra Cell, Newtown, CT, USA) equipped with a probe with 13 mm titanium tip (inserted to a depth of 3 cm). The temperature was controlled by using a water bath. The energy released in the system at the end of US treatment was about 15 kJ after 5 min, 33 kJ after 13 min, and 46 kJ after 23 min. After sonication, the conjugates coded as SYH-D and SYH-MD, respectively, were cooled in ice water to stop the reaction and further analysed. For samples exposed for 5 min to US treatment, no changes in volume sample have been recorded. For samples exposed for 13 min and 23 min to US, a water loss between 5–10% was measured. Thus, after cooling, distilled water was added to reach the initial volume. For each type of reaction mixture, the control was obtained without sonication by conventional heating, and the slurry was introduced in closed glass tubes at 70 °C for 100 min using a block heater (Stuart SBH200D, Essex, UK). The selection of the conventional heating conditions was considered based on the formation of the Maillard reaction compounds that, at a heating time lower than 100 min, did not occur.

### 2.4. Characterization of the Maillard Reaction Products

The pH of the conjugates was measured using a calibrated pH-meter (Lab 865, Xylem Analytics Germany, GmbH). The conjugates absorbance at 284 nm and 420 nm was recorded on diluted sample (0.4 mg/mL) using a UV-Vis spectrophotometer (Shimadzu UV-1900i, Kyoto, Japan), as described elsewhere [13]. The absorbance measured at 284 nm (A_284_) is an indicator of colourless intermediate compounds, while the absorbance measured at 420 nm (A_420_) indicate the content of advanced stage Maillard reaction products.

Glycation degree (GD) was measured according to the OPA method, as previously detailed [14]. The GD was calculated based on Equation (1):

(1) $$GD\;\left(\%\right)=\frac{A_{n}-A_{s}}{A_{n}}\times 100$$

where *A_n_* is the absorbance of SYH and *A_s_* represents the absorbance of the SYH:polysaccharide mixture after conjugation. 

The colour coordinates of the conjugated samples, expressed as lightness (L*), a* (+red; −green) and b* (+yellow; −blue), were measured by using the Chroma Meter CR-410 (Konica Minolta Sensing Americas Inc., Ramsey, NJ, USA) calibrated using a standard plate (L* = 93.01, a* = −0.11; b* = 4.68), and the standard illuminant C at an observed angle of 2o. The samples were prepared for the colour measurements by settling the loose powder through tapping using a clear glass window. The results are displayed as browning index (BI) and chroma (C*), values that were calculated with Equations (2) and (3) based on the L*, a*, and b* coordinates [3,15]:

(2) $$BI=\frac{\left(\frac{a^{*}\;+\;1.75\;\times \;L^{*}}{5.65\;\times \;L^{*}\;+\;a^{*}\;-\;3.01\;*\;b^{*}}\right)-0.31}{0.17}\times 100$$

C* = (a*^2^ + b*^2^)^1/2^ (3)

The ABTS^+^ radicals-scavenging activity (ABTS^+^-RSA) was measured as reported by Dumitrașcu et al. [16]. The results are expressed as µM Trolox equivalent/dry weight sample (µM TE/g d.w.).

### 2.5. Application of SYH-Based Maillard Conjugates as Carrier for Water Soluble Compounds

#### 2.5.1. Preparation of Anthocyanins Extract from BC Pomace

The BC pomace (seeds, pulp, and peel), resulted after juice separation by squeezing the fresh fruits, was freeze dried (CHRIST Alpha 1–4 LD plus, Germany) at −42 °C under a pressure of 0.10 mBar for 72 h. The freeze-dried pomace was stored under refrigeration in closed jars until use. For the preparation of the BC extract, the pomace was first grounded using a blade mill of 180 W electric power (Bosch, Stuttgart, Germany). The obtained powder was then mixed with 70% ethanol at a ratio of 1:32 (*w*/*v*). The mixture was sonicated for 30 min by using an ultrasonic bath (40 kHz, 100 W) at a temperature of 37 ± 2 °C. The sonicated sample was centrifuged at 9000 rpm for 5 min, and the supernatant was dried at 40 °C using a vacuum rotary evaporator (RVC 2–18, Martin Christ, Gefriertrocknungsanlagen GmbH, Osterode am Harz, Germany) before further use for encapsulation.

#### 2.5.2. Chromatographic Analysis of the BC Pomace Extract

The high-performance liquid chromatographic (HPLC) analysis of the anthocyanins from the BC samples was conducted on a Thermo Finnigan HPLC system (Thermo Scientific, Waltham, MS, USA). The separation and identification of the main anthocyanin compounds was employed on a Synergi 4u Fusion-RP 80A (150 × 4.6 mm, 4 μm) column, at the 520 nm wavelength, at an oven temperature of 27 °C. The samples were filtered through a C18 Sep-Pack cartridge-Waters and through a 0.22-μm syringe filters (Bio Basic Canada Inc., Toronto, ON, Canada). The mobile elution phase consisted of two main solvents that were 10% formic acid (A) and 100% methanol (B) with a vertical gradient. The injection volume was 10 µL at a flow rate of 1.0 mL/min. The identification and quantification of the compounds were made based on each compound’s calibration curve, and the results were expressed as mg/g d.w. 

#### 2.5.3. Encapsulation of Bioactive Compounds from Aronia

The freeze-dried SYH-MD and SYH-D conjugates obtained as presented above (Section 2.3) and HCW (prepared as showed in Section 2.2) were suspended individually in distilled water to reach a final concentration of 5% and stirred vigorously for efficient hydration. Six encapsulation variants were obtained by mixing the carrier solutions with aronia extract, as shown in Table 1 and Figure 1. All the samples were freeze dried. 

#### 2.5.4. Microcapsule Powders Characterization

The retention efficiency (RE) and encapsulation efficiency (EE) were assessed as reported elsewhere [17]. The total anthocyanins content (TAC) was expressed as mg cyanidin-3-glucoside/g dry weight (mg C3G/g d.w) [18]. The in vitro digestibility was evaluated as described by Dumitrașcu et al. [17] and expressed as the bioavailable portion of TAC according to Equation (4):

(4) $$Bioavailability\;\left(\%\right)=\frac{mg\;TAC\;after\;digestion}{mg\;TAC\;in\;microcapsule\;powder}\times 100$$

In brief, 100 mg of powder sample was mixed with 10 mL of Tris-HCl buffer (10 mM, pH 7.5) and transferred into simulated gastric juice (SGJ) at a ratio of 1:2 (mg/mL). The SGJ was obtained from porcine pepsin (Sigma Aldrich, USA) (40 mg/mL in 0.1 M HCl) at a final pH 2.0. After 2 h of incubation in SGJ, the resulting sample was mixed with simulated intestinal juice (SIJ) at a ratio of 1:1 (*v*/*v*) and incubated at 37 °C for another 2 h in an SI e300R orbital shaking incubator (Medline Scientific, Chalgrove, Oxon, UK), at 150 rpm. SIJ was obtained from 2 mg pancreatin of porcine pancreas/mL 0.9 M sodium bicarbonate at a final of pH 7.0. At the end of digestion, 0.2 mL of sample was used for the anthocyanins determination, as mentioned above.

The colour of the powders after freeze drying are presented based on L*, a*, b*, total colour change (Δ*E*) coordinates, and were collected using the Chroma Meter CR-410 (Konica Minolta Sensing Americas Inc., Ramsey, NJ, USA). ΔE was calculated as follows [10]:

(5) $$∆E=\sqrt{({L_{c}^{*}-L)}^{2}+({a_{c}^{*}-a^{*})}^{2}+({b_{c}^{*}-b^{*})}^{2}\;}$$

where$\;L_{c}^{*}$, $a_{c}^{*}$, and $b_{c}^{*}$ represent the colour coordinates of control sample coded as SYH.

The storage stability of the microcapsule powders was tested at 20 °C and was reported as relative concentration (%) of TAC and ABTS^+^-RSA measured after 90 days of storage, in respect to the values measured immediately after encapsulation. The morphology of the microcapsules powders was evaluated by using a scanning electron microscope (SEM) (QUANTA 200, FEI/Thermo Fisher, Hillsboro, Oregon, USA) at two magnifications of 1000× and 5000×. The secondary electrons signal emitted by the surface atoms excited using the incident beam accelerated at 19 kV potential was selected to improve the scanning resolution. 

### 2.6. Statistical Analysis

The results of replicated experiments are expressed as mean followed by standard deviation, calculated from at least duplicate samples. The number of determinations performed for each investigated parameter is provided in the results and discussion part. The statistical differences between samples were tested based on an ANOVA parametric test, after passing normality (Ryan Joiner test) and homoscedasticity test (Levene test) at 0.05 significance level. Post hoc analysis based on a Tukey test at 95% confidence was performed at *p*-values lower than 0.05 resulted in ANOVA method. The software used to perform the statistical calculations was Minitab19 (Minitab Inc., State College, PA, USA). 

## 3. Results and Discussion

### 3.1. Conjugate Characterization

#### 3.1.1. pH

The pH of the samples was more influenced by the conjugation method and less by the polysaccharide type used in the Maillard reaction (Table 2). Compared to SYH:polysaccharide mixture before glycation, US treatment decreased the pH during the Maillard reaction. Usually, the Maillard reaction leads to the formation of organic acids, such as formic and acetic acids, and the reduction in the amino-groups of the proteins/peptides with consequences on the pH that decreases [19]. After 5 min of US exposure, the pH reached a minimum in both samples, while further exposure led to the pH increase, an indication of peptides denaturation that favoured the exposure of hidden amino groups causing the increase of pH [6]. Compared to conventional heating, the pH values of the US conjugated samples were slightly lower indicating the higher Maillard reaction rate when using US. Abdelhedi et al. [15] investigated the effect of the US pre-treatment on the progression of the Maillard reaction between smooth-hound viscera peptides with low molecular weight and sucrose. The authors reported that the pH of the conjugated sample pre-treated with ultrasound increased during the first 60 min of heat treatment at 90 °C and then decreased. The pH increase was associated with the hydrolysis of intact peptides at thermal treatment coupled to the progressive exposure of hidden amino groups. Our results are in line with those reported by Zhang [20] who investigated the Maillard conjugation of whey protein isolate hydrolysate with galactose by conventional wet method. The authors reported that the pH of the conjugate did not vary significantly after 4 h of heating time due to amino groups produced by enzymatic hydrolysis that prevented pH reduction.

#### 3.1.2. Glycation Degree (GD)

In conventional heating, polysaccharide type did not exert any influence on GD, while during US treatment, the GD of the samples reached a maximum after 5 min. of ultrasonication and was more dependent on the polysaccharide type. Thus, the GD of the conjugate prepared with dextran was higher by about 13% than the SYH:MD conjugate (Table 2). US treatment for 5 min was able to provide a similar GD with what conventional heating was able to reach it after 100 min, most probably due to acoustic cavitation that accelerated the molecular motion of the proteins, leading to the disruption of quaternary and tertiary structure of protein, which in the end promoted the exposure of reactive groups [6,21]. In addition, the energy that resulted during US exposure accelerated the grafting between reactants, as the reactive groups of SYH and polysaccharides were in closer proximity [21]. On the other hand, increasing the exposure to ultrasound up to 23 min caused the decrease of GD by about 38% in both conjugated samples. This effect was associated with protein unfolding caused by excessive ultrasound, that, through increased hydrophobic interactions and/or protein–protein hydrogen bonding, re-embedded the exposed amino acid, inhibiting the Maillard reaction [13,22]. In another study, the decrease in the grafting degree was attributed to the degradation or polymerization of reducing sugars [23]. Our results are in line with those reported by Liu et al. [24] for whey protein hydrolysate conjugated with galactose. The exposure to US had a negative effect on GD; however, in contrast with our study, the GD decreased, regardless of the US exposure time [22]. The authors attributed the decrease to increasing the free amino groups content due to the heat-driven exposure of inherently hidden domains. 

#### 3.1.3. UV-Absorbance and Browning Intensity Measurement of the Maillard Conjugates

The absorbance of the conjugated samples was affected by the glycation method, conventional heating resulting in significant higher values of the UV-absorbance than US treatment (Table 2). Polysaccharide type had a significant effect on the absorbance measured at 420 nm only for the conjugates obtained by conventional heating, indicating the higher ability of dextran to promote the formation of advanced stage Maillard reaction products. Similar to our study, Zhao et al. [25] found that, compared to conventional heating, US resulted in the decrease of the browning degree of Maillard conjugates based on soy proteins due to US’ ability to inhibit the polymerization of intermediate products during the Maillard reaction. Moreover, our results are in line with the study regarding the whey protein hydrolysate:galactose conjugates prepared by using the US treatment for up to 60 min at 90 °C [24]. The authors reported that the whey peptides conjugates absorbance at 294 nm and 420 nm was not affected by US exposure. A possible explanation was provided by Nooshkam and Madadlou [26], who indicated that the conjugation of proteins with sugars is more intense than with peptides, as they have the potential of forming large conjugates with sugars while presenting lower reactivity than peptides.

The colour changes of the Maillard conjugates prepared with conventional and US treatment, expressed as BI and Chroma (C*), are summarized in Table 2. BI and C* values were dependent on both polysaccharide type and glycation method. US treatment was able to provide higher BI and C* values compared to conventional heating. BI and C* values of the conjugates increased significantly (*p* < 0.05) with US exposure time, the highest values being recorded after 23 min of US treatment. Moreover, conjugates prepared with dextran showed increased browning intensity compared to conjugates prepared with maltodextrin, indicating that US promotes to a higher extent the formation of brown compounds in SYH:D sample. 

#### 3.1.4. Antioxidant Activity

**Figure 2:**
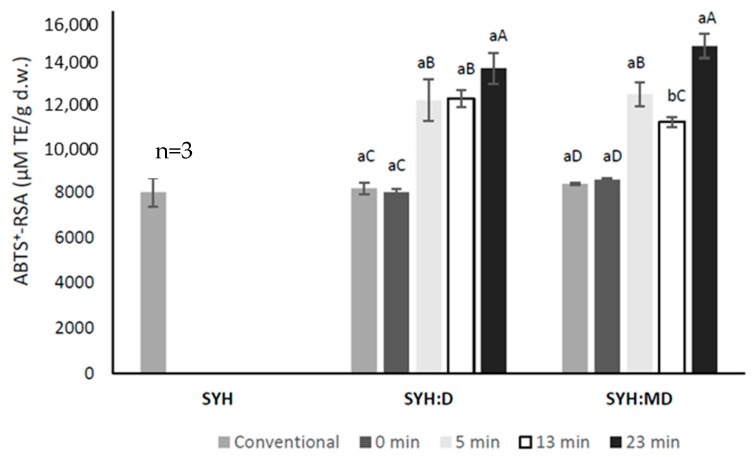
ABTS^+^-RSA of the spent yeast protein hydrolysate (SYH) and SYH conjugated with dextran (D) and maltodextrin (MD) with US treatment or conventional heating (70 °C, 100 min). n—number of determinations. Samples that, for each mixture, do not share the same lowercase letter (a, b) are statistically significant at *p* < 0.05, based on Tukey post hoc test. Samples that, for the same ultrasound time, do not share the same uppercase letter (A, B, C, D) are statistically significant at *p* < 0.05, based on Tukey post hoc test.

The antioxidant activity of the Maillard conjugates was measured based on the ABTS^+^-RSA, the results being depicted in Figure 2. The treatments applied for obtaining the Maillard conjugates resulted in no new peptides forming. Because of the short time and supplemented energy during treatment, only some local unfolding events could occur in the protein molecules. Therefore, the antioxidant activity increase was assigned to the Maillard reaction products obtained through the interaction between the yeast protein hydrolysates and carbohydrates. The US treatment exerted a positive effect on the antioxidant potential, generating higher values compared to conventional heating, and was less affected by the carbohydrate type. The highest antioxidant activity was calculated after 23 min of US exposure in the SYH:MD conjugate (14454 ± 300 µM TE/g d.w.). The ABTS^+^ radical scavenging activity of the Maillard conjugates during conventional heating (70 °C, 100 min) did not vary compared to peptide:carbohydrate mixture prior to conjugation assisted by ultrasonication, indicating the positive effect of US treatment on increasing the antioxidant activity. Our results are in line with previously reported studies. Liu et al. [24] reported that the ABTS^+^ radical scavenging activity of the whey proteins/peptides:galactose Maillard conjugates was enhanced with increasing the exposure to ultrasound, reaching a maximum after 60 min of US treatment. In addition, Abdelhedi et al. [15] showed that US pre-treatment of the low molecular weight peptides with sucrose contributed to enhancing the reducing power of the resulting conjugates. On the other hand, the Maillard reaction products obtained from hydrolysate (derived from mussel meat) glucose mixture using US pre-treatment followed by conventional heating exhibited a higher antioxidant activity compared to heat treatment or US treatment [27]. 

### 3.2. Chromatographic Profile of Black Chokeberries Pomace

**Figure 3:**
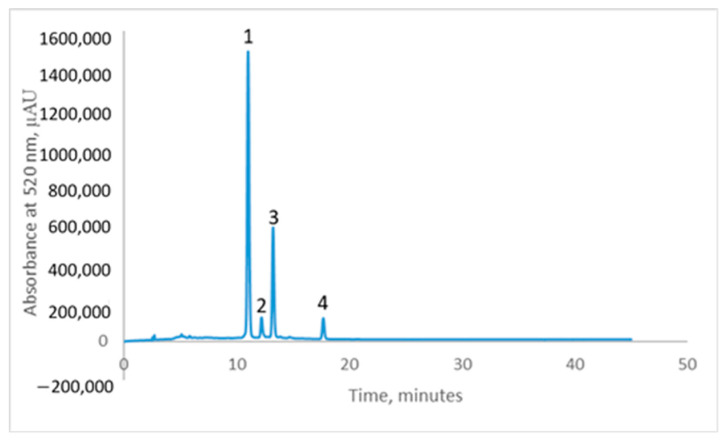
The chromatographic profile of BC pomace extract (B)—Peak 1—cyanidin-3-*O*-galactoside, Peak 2—cyanidin-3-*O*-glucoside, Peak 3—cyanidin-3-*O*-arabinoside and Peak 4—cyanidin-3-*O*-xyloside.

The chromatographic profile of the pomace extract (Figure 3) revealed the presence of four main anthocyanins, namely cyanidin-3-*O*-galactoside (24.721 ± 0.816 mg/g d.w.), cyanidin-3-*O*-glucoside (1.712 ± 0.010 mg/g d.w.), cyanidin-3-*O*-arabinoside (8.901 ± 0.225 mg/g d.w.), and cyanidin-3-*O*-xyloside (1.824 ± 0.007 mg/g d.w.). The results are consistent with the studies reported in the scientific literature that assessed the phytochemical composition of different samples of *Aronia melanocarpa* [28,29].

### 3.3. Microcapsules Characterization

#### 3.3.1. Retention Efficiency and Encapsulation Efficiency

During the Maillard reaction, the available nucleophilic residues of proteins, like cysteine and tyrosine, interact with the carboxylic groups of polysaccharides, providing reaction sites for anthocyanins encapsulation [30]. The encapsulation process used as carrier materials for anthocyanins encapsulation SYH:D or SYH:MD and HCW. The addition of HCW was decided based on previous studies that indicated the potential of yeast cell wall as encapsulating material, due to the high content of proteins and β-glucan [3,31]. MD is one of the most used wall material for anthocyanins encapsulation, whereas the anionic polysaccharides, like dextran, provide superior protection for anthocyanins from aronia extract through electrostatic interaction with flavylium cation of anthocyanins [10]. In this study, the RE and EE of anthocyanins (Table 3) were more influenced by the addition of HCW than by Maillard conjugation. The sample containing only HCW (V2) showed, in the same time, the highest RE (66.00 ± 2.58%) and the lowest EE (67.09 ± 2.26%), indicating the positive effect on retention and the negative effect on encapsulation. The addition of HCW in V4 and V6, increased the RE by about 11% and 9%, respectively (compared to V3 and V5, respectively), while for EE, HCW did not play a significant effect on improving anthocyanins encapsulation. When compared to the control sample (SYH), the Maillard conjugates did not show significant improvement on encapsulation efficiency. Moreover, the HCW sample was able to encapsulate about 20% fewer anthocyanins than the SYH- and SYH-based conjugates. The lower EE of HCW is attributed to the enzymatic hydrolysis that resulted in increasing the cell permeability, while decreasing the loading of bioactive compounds [32]. It seems that the presence of mannoproteins in the HCW responsible for the porosity of the cell wall favoured the retention of anthocyanins, but were not able to provide superior encapsulation efficiency [33]. Our results are higher than those reported by Ahmad et al. [34], who calculated a RE of the phenolic compounds of about 50% for microparticles obtained with β-glucan. Compared to our results, active yeast was found to encapsulate lower levels of anthocyanins [35], whereas yeast hulls were able to encapsulate the anthocyanins from *Hibiscus sabdariffa* for up to 40% [23]. Kurek et al. [5] investigated the microencapsulation of anthocyanins from chokeberries with plasmolysed yeast cells of various species. The EE was dependent on the type of yeast cells, the maximum value (about 54%) being obtained when using yeasts from the bottom and top fermentation of the beer. Marson et al. [3] microencapsulated ascorbic acid by combining hydrolysed spent brewer’s yeast cell debris and maltodextrin through Maillard conjugation. The authors concluded that hydrolysed cell debris of spent brewer’s yeast is an excellent material for the encapsulation of bioactives. Our results are comparable with those reported for other sources of protein hydrolysates used in combination with the Maillard conjugation for preparing carrier materials for the delivery of bioactive compounds [36].Overall, the EE of the TAC was higher compared to those of total phenolic compounds (TPC), regardless of the combination of wall materials (Table S1). As shown in Table S1, the highest RE of TPC was measured in V1 (69.95 ± 2.21 %) and the lowest in V2 (46.87 ± 0.4 %). The potential of HCW to retain TAC was higher by about 30% than of TPC. Regarding the TPC retention, the addition of HCW had a positive influence in V4 and a slightly negative effect on V6. For SYH:D conjugate, the addition of HCW did not contribute to increasing the TPC encapsulation.

**Figure 4:**
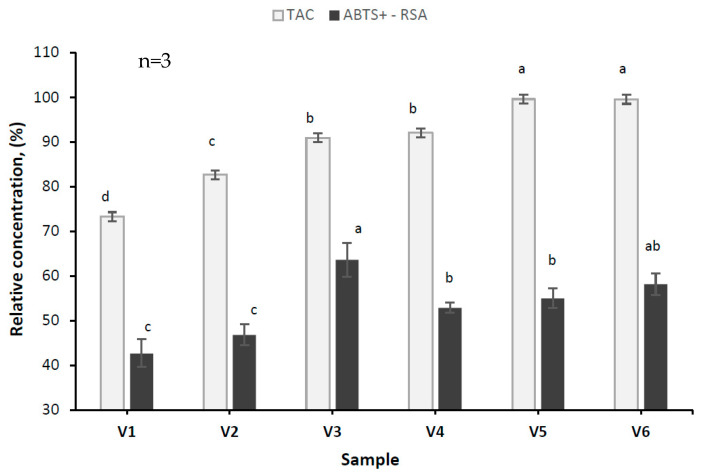
Stability at the end of 90 days of storage of TAC and ABTS^+^-RSA. TAC—total anthocyanins content, ABTS^+^-RSA—ABTS^+^ radical scavenging activity. n—number of determinations. Mean values that for the same parameter do not share the same lowercase letter (a, b, c, d) are statistically significant at *p <* 0.05, based on Tukey post hoc test.

The stability during storage was monitored for 90 days at 20 °C. The results expressed as relative concentration of TAC, and antioxidant activity (ABTS^+^-RSA) are depicted in Figure 4. The stability decreased during storage, significant variations (*p <* 0.05) being recorded between tested variants. For anthocyanins, the lowest stability was measured in the controls and V1 and V2 samples, while the highest was measured in the conjugated samples. These results indicate the positive effect of the Maillard reaction in providing superior protection of anthocyanins during storage. Regardless of the HCW presence, SYH:D and SYH:MD conjugates were able to offer efficient protection to anthocyanins of 99% and 91%, respectively. The protection of TPC during storage ranged between 74% in V2 to 91% in V3 (Figure S1). A better stability of TPC was noticed when using SYH:MD conjugates for encapsulation. However, the addition of HCW decreased the stability of TPC to about 73% (Figure S1). Regarding the ABTS^+^-RSA (Figure 4), the stability in conjugated samples ranged between 55% and 63%, whereas the stability for control samples ranged between 42% and 46%. Thus, the Maillard conjugation assisted by US promoted the increased stability of the ABTS^+^-RSA. Similar results were reported recently by Parandi et al. [36] for sesame protein hydrolysate:gum Arabic conjugates, loaded with anthocyanins. After 90 days of storage, the TAC, and antioxidant activity in powders prepared with Maillard conjugates decreased by about 7.8%, and 7.36%, respectively, the decrease being by 50% lower than the corresponding sample based only on sesame protein hydrolysate. 

#### 3.3.2. Water Activity (a_w_) 

The dry matter content was higher than 94% for all tested samples. The a_w_ values of the microcapsule powders during storage at different temperatures are presented in Table 3. The a_w_ was dependent on the carrier material used for encapsulation, the lowest value being recorded in the sample that used SYH:MD conjugate and HCW (0.166 ± 0.001), whereas the highest was measured in V5 (SYH:D). Lower a_w_ values, with the addition of maltodextrin as carrier material, were also reported by Velez-Erazo et al. [37] and Akbarbaglu et al. [38]. The addition of HCW in V4 decreased the a_w_ value by about 13% when compared to the sample without HCW (V3), and had no influence on V6 (when compared to V5). Compared to V1, the use of dextran in the mixture decreased the a_w_ of V5 and V6. Regardless, the a_w_ of all samples was lower than 0.3 over the entire tested period. This a_w_ is a threshold limit under which the powders are stable, limiting the microbial growth and the susceptibility to enzymatic and non-enzymatic processes during storage [3,17].

#### 3.3.3. Colour Coordinates

Colour represents an important property of food products. The images of the investigated microcapsule powders are presented in Figure 1. In terms of lightness (L*), the lowest value was measured in the sample that used as carrier SYH (49.35 ± 0.01) and the highest was measured in the sample containing SYH:D Maillard conjugate (56.94 ± 0.01) (Table 3). The type of carbohydrate used in the Maillard conjugation influenced (*p* < 0.05) the L* coordinate, generating higher lightness compared to the corresponding control samples. However, the addition of HCW in V4 and V6 decreased the L* coordinate compared to samples without HCW. Regarding the a* coordinate (Table 3), the lowest value was calculated in V1 and V2, while the highest in V3 and V5, indicating a more intense red colour of V3 and V5 samples. Similar to L* coordinate, the red colour was negatively influenced by the HCW addition. Lower a* values were measured on samples containing HCW. The results are lower than those reported by Kurek et al. [5], who encapsulated anthocyanins from chokeberries using different species of yeasts, and higher than those reported by Nguyen et al. [33] for anthocyanins encapsulation using yeast hulls. The differences were associated with the encapsulation method and the yeast strains used as carrier material. As presented in Table 3, the b* coordinate showed significant differences between samples. Positive values registered for V1–V4 variants indicate the yellow tendency, whereas the negative values measured in V5–V6 suggest a more tendency to blue colour. Compared to control samples (V1 and V2), the use of SYH:MD or SYH:D conjugates resulted in the decrease in the b* coordinate. Moreover, the addition of HCW in the SYH:MD mixture caused the decrease in the b* coordinate by about 53% compared to SYH:MD sample without HCW. Unlike the SYH:MD-based samples, the addition of HCW in the SYH:D sample increased the b* coordinate by about 18%. The ΔE values presented significant differences (*p* < 0.01) between tested variants, indicating the strong effect of the Maillard conjugation on the wall materials used for anthocyanins encapsulation.

#### 3.3.4. In Vitro Digestibility

**Figure 5:**
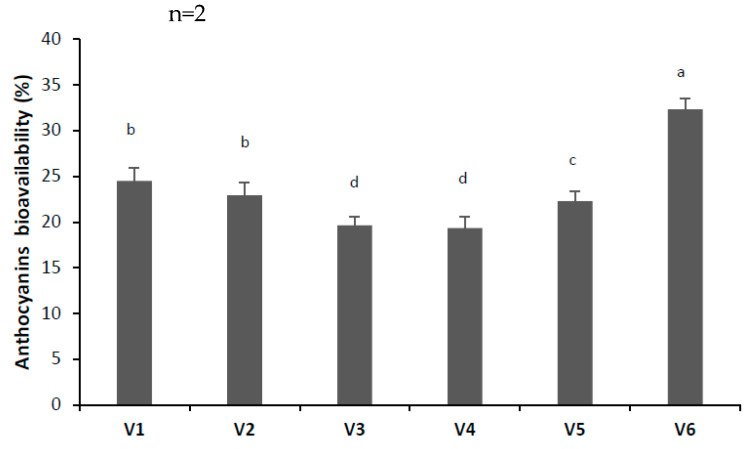
The bioavailability of anthocyanins at the end of the simulated gastrointestinal digestion. n—number of determinations. Mean values that do not share the same lowercase letter (a, b, c, d) are statistically significant at *p* < 0.05, based on Tukey post hoc test.

The bioavailability of the encapsulated anthocyanins after 4 h of simulated gastrointestinal digestion is depicted in Figure 5. The degradation of anthocyanins under gastrointestinal conditions was dependent on the wall material. The highest bioavailability of anthocyanins was observed in V6, indicating that the SYH:D and HCW mixture is suitable for providing anthocyanins protection (Figure 5). When compared with control samples (V1 and V2), the bioavailability of TAC in V3 and V4 samples was lower, indicating that the Maillard conjugation affected the anthocyanins’ protection provided through encapsulation during gastrointestinal digestion. The addition of HCW on the SYH:D conjugate allowed retaining by about 40% more anthocyanins than the conjugate without HCW, indicating this combination of wall material as having a good potential to reduce the degradation in the gastrointestinal tract. Similar results were reported by Ahmad et al. [34], who encapsulated anthocyanins using β-glucan as a wall material. Although maltodextrin was reported as having excellent potential for protecting anthocyanins degradation during digestion [39], it seems that the Maillard conjugation in this combination was not able to provide superior protection. Other studies suggested that maltodextrins have good encapsulation properties when used in admixture with other gums [10]. The bioavailability of anthocyanins reported in our study is lower compared with the results reported by Jang and Koh [40], who tested the effect of anthocyanins and chlorogenic acid isomers encapsulation on the in vitro digestion. The authors encapsulated the aronia extract by using a combination of polysaccharides, and found a bioavailability of about 75% for anthocyanins after simulating the intestinal digestion. Anthocyanins are unstable at high pH, as the shift from the acidic stomach conditions to the neutral pH in the duodenum causes the hydrolysis and/or degradation [41]. 

#### 3.3.5. Microstructure of the Microcapsule Powders

**Figure 6:**
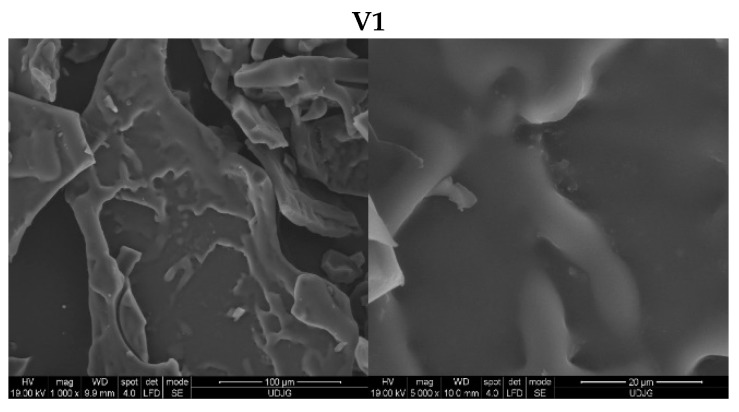

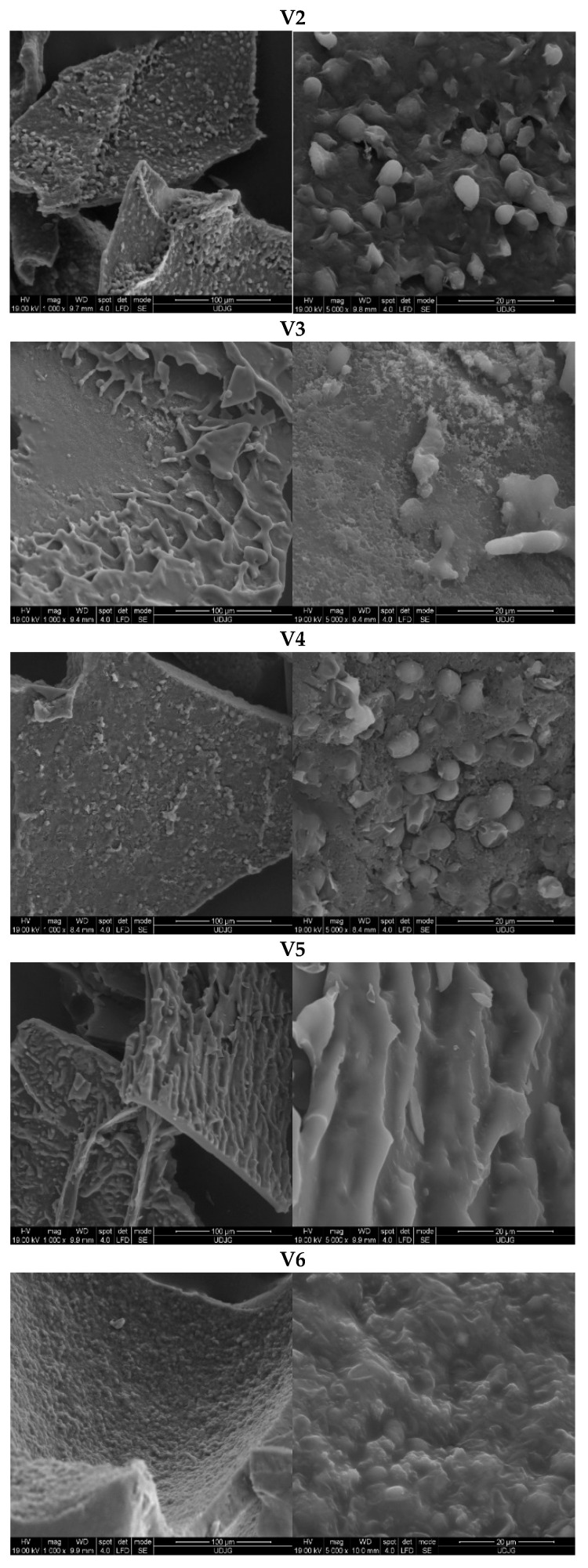
SEM analysis of the microcapsule powders including aronia phenolic compounds and SYH (**V1**), HCW (**V2**), SYH:MD (**V3**), SYH:MD + HCW (**V4**), SYH:D (**V5**), SYH:D + HCW (**V6**) as carriers. SYH—spent yeast protein hydrolysate, HCW—hydrolysed cell wall, MD—maltodextrin, D—dextran.

The microstructure of the tested microcapsule variants is presented in Figure 6. The morphology of the microcapsules was specific to samples obtained by freeze drying. Thus, most of the tested variants presented irregular, and glassy or flake-like structures [10]. The structural rigidity can be attributed to the lack of water in the liquid state during sublimation that at the end protect the anthocyanins against heat and oxygen exposure [42,43]. The Maillard conjugation and the combination of carriers produced variations between samples. The surface of SYH based sample (V1) presented an amorphous, flat, and tight surface, specific to powders including hydrolysed proteins. The Maillard conjugation of SYH with MD or D lead to the formation of a rough compressed surface because of the polysaccharides interaction with proteins, which reduced aggregation of the proteins [44]. The morphology of the variants containing HCW was characterized by a higher level of agglomeration, where some of the cells were torn or half opened, the structure appearing like a honeycomb. The morphology is the result of the enzymatic hydrolysis that decreased the rigidity of the cell wall [45]. The observations regarding the morphology of the microcapsule powders are in agreement with other reported studies that used yeast cells for anthocyanins encapsulation [5].

## 4. Conclusions

The study explored, for the first time, the potential of brewer’s spent yeast protein hydrolysate to produce Maillard conjugates when combined with maltodextrin or dextran, to be used for the encapsulation of the anthocyanins from aronia pomace. The use of ultrasound for obtaining Maillard conjugates ensured the increase in the antioxidant activity of the tested conjugates by about 50% when compared to non-conjugated samples. The Maillard conjugates presented good ability to encapsulate bioactive active compounds from aronia, the highest encapsulation efficiency being recorded for samples that used maltodextrin as polysaccharide in the Maillard reaction. Although Maillard conjugation assisted by ultrasound did not contribute significantly to increasing the encapsulation potential of spent yeast hydrolysate, the positive contribution was revealed during storage, where powders prepared with Maillard conjugates presented enhanced stability. The Maillard conjugation played a positive effect on the stability of the anthocyanins during storage, more than 90% of anthocyanins being protected after 90 days of storage. The best protection of anthocyanins during in vitro digestion was recorded in the spent yeast hydrolysate:dextran conjugates with or without the hydrolysed cell wall. In conclusion, our study highlighted that the enzymatic hydrolysis combined with the Maillard reaction assisted by ultrasound has a great potential on valorisation of spent brewer’s yeast by obtaining encapsulating materials for loading bioactive compounds that can be added successfully into a food product. 

## Figures and Tables

**Table 1:** Sample codifications and protocol used for obtaining microcapsule powders.

Sample Code	Carriers (g Carrier/100 g Water)	Ratio between Carriers *w*/*w*	Conjugation Assisted by US before Encapsulation
V1	SYH (5)	Not applicable	No
V2	HCW (5)	Not applicable	No
V3	SYH (2.5) MD (2.5)	1:1	Yes
V4	SYH (2.5) MD (2.5) HCW (5)	1.5:1.5:1	Yes, SYH:MD mixture
V5	SYH (2.5) D (2.5)	1:1	Yes
V6	SYH (2.5) D (2.5) HCW (5)	1.5:1.5:1	Yes, SYH:D mixture

SYH—spent yeast protein hydrolysate, HCW—hydrolysed cell wall, MD—maltodextrin, D—dextran.

**Table 2:** Changes in pH, glycation degree (GD), absorbances at 284 nm (A_284 nm_) and 420 nm (A_420 nm_), and colour properties (BI—browning index and C*—chroma) of spent yeast protein hydrolysate (SHY) conjugates with dextran (D) and maltodextrin (MD) at different ultrasound time. n = 3.

Conjugate	Parameter	Conventional Heating (70 °C, 100 min)	Ultrasound Time (min)			
			0	5	13	23
SYH:D	pH	6.80 ± 0.01 ^bA^	6.96 ± 0.02 ^aA^	6.66 ± 0.02 ^dA^	6.72 ± 0.01 ^cB^	6.74 ± 0.01 ^cA^
	GD%	30.75 ± 2.1 ^aA^	-	33.89 ± 0.56 ^aA^	22.37 ± 0.78 ^bA^	21.25 ± 1.94 ^bA^
	A_284 nm_	3.04 ± 0.21 ^aA^	2.68 ± 0.006 ^bA^	2.64 ± 0.01 ^bcA^	2.51 ± 0,15 ^bA^	2.66 ± 0,03 ^bA^
	A_420 nm_	0.148 ± 0.004 ^aA^	0.102 ± 0.001 ^bA^	0.109 ± 0.001 ^bA^	0.093 ± 0.001 ^cA^	0.102 ± 0.009 ^bA^
	L*	33.35 ± 0.03 ^aA^	33.20 ± 0.01 ^bA^	32.91 ± 0.03 ^cA^	32.16 ± 0.06 ^dB^	32.12 ± 0.04 ^dA^
	a*	0.21 ± 0.04 ^cA^	0.36 ± 0.01 ^bA^	0.33 ± 0.02 ^bA^	0.51 ± 0.04 ^aA^	0.58 ± 0.03 ^aA^
	b*	1.42 ± 0.05 ^bA^	−0.26 ± 0.01 ^dA^	1.35 ± 0.02 ^cA^	1.41 ± 0.01 ^bA^	1.71 ± 0.03 ^aA^
	BI%	4.52 ± 0.16 ^cA^	-	4.64 ± 0.04 ^cA^	5.37 ± 0.08 ^bA^	6.47 ± 0.18 ^aA^
	C*	4.12 ± 0.31 ^bA^	0.407 ± 0.04 ^dA^	3.86 ± 0.11 ^cA^	4.53 ± 0.07 ^bA^	6.52 ± 0.31 ^aA^
SYH:MD	pH	6.78 ± 0.01 ^cA^	6.96 ± 0.02 ^aA^	6.68 ± 0.01 ^dA^	6.86 ± 0.01 ^bA^	6.68 ± 0.01 ^dB^
	GD%	30.05 ± 0.27 ^aA^	-	29.54 ± 1.62 ^aB^	24.04 ± 1.08 ^bA^	18.50 ± 0.91 ^cA^
	A_284 nm_	2.89 ± 0.21 ^aA^	2.66 ± 0.02 ^aA^	2.51 ± 0.14 ^aA^	2.63 ± 0.04 ^aA^	2.66 ± 0.02 ^aA^
	A_420 nm_	0.129 ± 0.004 ^aB^	0.101 ± 0.001 ^bA^	0.094 ± 0.001 ^cB^	0.091 ± 0.001 ^cA^	0.103 ± 0.01 ^bA^
	L*	33.42 ± 0.02 ^aA^	33.25 ± 0.03 ^bA^	33.08 ± 0.05 ^cA^	32.71 ± 0.25 ^cdA^	32.06 ± 0.06 ^eA^
	a*	0.18 ± 0.005 ^dA^	0.36 ± 0.02 ^bA^	0.24 ± 0.05 ^cA^	0.44 ± 0.06 ^aA^	0.49 ± 0.01 ^aB^
	b*	0.81 ± 0.02 ^dB^	−0.27 ± 0.01 ^eA^	1.27 ± 0.04 ^cA^	1.40 ± 0.01 ^bA^	1.50 ± 0.06 ^aB^
	BI	2.65 ± 0.05 ^dB^	^-^	4.20 ± 0.24 ^cB^	5.07 ± 0.17 ^bB^	5.63 ± 0.17 ^aB^
	C*	1.40 ± 0.06 ^dB^	0.41 ± 0.02 ^eA^	3.38 ± 0.28 ^cB^	4.31 ± 0.10 ^bB^	5.02 ± 0.38 ^aB^

n—number of determinations. Samples for the same row that do not share the same lowercase letter are statistically significant at *p <* 0.05, based on Tukey post hoc test. Samples for the same column and parameter that do not share the same uppercase letter are statistically significant at *p <* 0.05, based on Tukey post hoc test.

**Table 3:** Retention efficiency (RE) and encapsulation efficiency (EE), and water activity (a_w_) and colour indices (L*, a*, b*, ΔE) of microcapsules embedded with aronia anthocyanins. n = 3.

Sample Code	RE (%)	EE (%)	a_w_	L*	a*	b*	ΔE
V1	61.92 ± 2.88 ^b^	81.22 ± 3.79 ^b^	0.233± 0.021 ^a^	49.35 ± 0.01 ^f^	6.65 ± 0.02 ^e^	2.3 ± 0.01 ^a^	-
V2	66.00 ± 2.58 ^a^	67.09 ± 2.26 ^c^	0.190± 0.001 ^a^	50.66 ± 0.01 ^e^	5.07 ± 0.02 ^f^	1.23 ± 0.0 ^b^	2.31 ± 0.01 ^e^
V3	58.25 ± 0.38 ^bc^	84.72 ± 2.56 ^a^	0.190± 0.006 ^a^	52.14 ± 0.00 ^c^	11.54 ± 0.01 ^b^	0.307 ± 0.005 ^c^	5.98 ± 0.02 ^b^
V4	64.44 ± 2.98 ^a^	88.72 ± 0.33 ^a^	0.166 ± 0.001 ^a^	51.1 ± 0.31 ^d^	8.94 ± 0.07 ^c^	0.145 ± 0.035 ^d^	3.56 ± 0.17 ^d^
V5	60.08 ± 2.79 ^b^	83.77 ± 2.53 ^a^	0.282 ± 0.004 ^a^	56.94 ± 0.01 ^a^	11.7 ± 0.02 ^a^	−0.17 ± 0.01 ^f^	9.44 ± 0.01 ^a^
V6	65.25 ± 2.21 ^a^	80.21 ± 2.59 ^b^	0.271 ± 0.012 ^a^	53.89 ± 0.01 ^b^	8.58 ± 0.01 ^d^	−0.14 ± 0.01 ^e^	5.51 ± 0.01 ^c^

n—number of determinations. Means that for the same column labelled with different lowercase letter (a–f), are statistically significant, based on 95% Tukey method.

## Data Availability

The original contributions presented in the study are included in the article, further inquiries can be directed to the corresponding author.

## Supplementary Materials

The following supporting information can be downloaded at: https://www.mdpi.com/article/10.3390/antiox13050570/s1, Table S1: Retention efficiency (RE %) and encapsulation efficiency (EE %) of TPC in microcapsules; Figure S1: Stability at the end of 90 days of storage of TPC. Mean values that do not share the same lowercase letter (a, b, c, d) are statistically significant at *p* < 0.05, based on Tukey post hoc test.
