# Supplementary material for: Ultrasound-Assisted Maillard Conjugation of Yeast Protein Hydrolysate with Polysaccharides for Encapsulating the Anthocyanins from Aronia
Source: Antioxidants (Basel). 2024 May 5;13(5):570. doi: 10.3390/antiox13050570 (PMC11117535; doi:10.3390/antiox13050570)
Supplement: Supplementary file 1 [file antioxidants-13-00570-s001.zip › antioxidants-2957526-supplementary.pdf]

SUPPLEMENTARY MATERIAL

Microcapsule powders characterization

Total phenolic compounds

The retention (RE) and encapsulation efficiency (EE) of phenolic compounds were determined as reported elsewhere by Dumitrașcu et al. (2021). The content of total phenolic compounds (TPC) was expressed as mg acid galic equivalent/ g dry weight (mg GAE/g d.w.). The *in vitro* digestibility was evaluated as described by Dumitrașcu et al. (2021), and expressed as bioavailability portion of TPC according to Eq (4) (Fredes et al., 2018).

$$Bioaccesibility\ (\%) = \frac{mg\ TPC\ after\ digestion}{mg\ TPC\ in\ microcapsule\ powder} \times 100 \quad (1)$$

The RE and EE of TPC are collected in **Table S1**. The highest RE of TPC was measured in V1 (69.95 ± 2.21 %) and the lowest in V2 (46.87 ± 0.4 %). Similar results were reported by Ahmad et al. (2018), who calculated a RE of the phenolic compounds of about 50% for microparticles obtained with β-glucan. Our results indicated that the HCW potential to retain TAC was higher by about 30% than of TPC. Regarding the TPC retention, the addition of HCW had a positive influence in V4 and a slightly negative effect on V6. For SYH:D conjugate, the addition of HCW did not contribute to increasing the TPC encapsulation (**Table S1**). Similar results were noticed for TPC encapsulation, where the lowest EE value was measured in the HCW sample (63.55 ± 0.91 %). Compared to V1, the use of Maillard conjugates did not change significantly the TPC encapsulation. Marson et al. (2020) microencapsulated ascorbic acid by combining hydrolyzed spent brewer's yeast cell debris and maltodextrin through Maillard conjugation. The authors concluded that hydrolyzed cell debris of spent brewer's yeast is an excellent material for bioactives encapsulation. Our results are comparable with those reported for other sources of protein hydrolysates used in combination with Maillard conjugation for preparing carrier materials for bioactive compounds delivery (Parandi et al., 2024). Overall, the EE of the TAC was higher compared to those of TPC, regardless of the combination of wall materials.

Table S1. Retention efficiency (RE %) and encapsulation efficiency (EE %) of TPC in microcapsules

Sample code	TPC	
	RE (%)	EE (%)
V1	69.95 ± 2.21^{aA}	75.08 ± 0.96^{cB}
V2	46.87 ± 0.4^{eB}	63.55 ± 0.91^{eB}
V3	52.17 ± 1.28^{cB}	76.74 ± 0.49^{cB}
V4	58.09 ± 1.20^{bB}	77.54 ± 0.54^{bB}
V5	52.65 ± 0.36^{cB}	78.47 ± 0.23^{aB}
V6	50.71 ± 0.36^{dB}	76.30 ± 0.72^{cB}

Means that for the same column labelled with different lowercase letter (a-f), are statistically significant, based on 95% Tukey method.

The stability during storage was monitored for 90 days at 25°C, and the results expressed as relative concentration of TPC are presented in **Figure S1**. The stability decreased during storage, significant variations ($p < 0.05$) being recorded between tested variants. The protection of TPC during storage ranged between 74% in V2 to 91% in V3. A better stability of TPC was noticed

when using SYH:MD conjugates for encapsulation. However, the addition of HCW decreased the stability of TPC to about 73%.

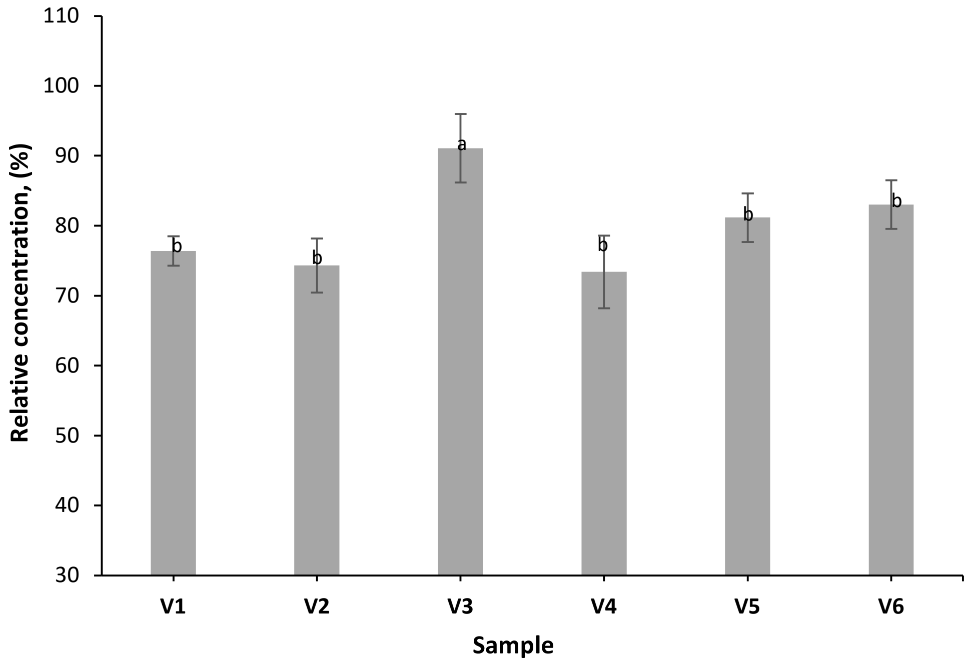

Figure S1. Stability at the end of 90 days of storage of TPC. Mean values that do not share the same lowercase letter (a, b, c, d) are statistically significant at $p < 0.05$, based on Tukey post hoc test.
